# Hereditary angioedema: A rare cause of recurrent abdominal pain

**DOI:** 10.12669/pjms.305.5524

**Published:** 2014

**Authors:** Xi Chen, Ying Xue Yang, Yu Lan Liu, Hua Tian Gan, Zhong Hui Wen

**Affiliations:** 1 Xi Chen, PhD, Department of Gastroenterology, West China Hospital, Sichuan University, Chengdu, Sichuan 610041, People’s Republic of China.; 2 Ying Xue Yang, MD, Department of Gastroenterology, West China Hospital, Sichuan University, Chengdu, Sichuan 610041, People’s Republic of China.; 3 Yu Lan Liu, MSc, Department of Gastroenterology, West China Hospital, Sichuan University, Chengdu, Sichuan 610041, People’s Republic of China.; 4 Hua Tian Gan, MD, Department of Gastroenterology and Geriatrics Medicine, West China Hospital, Sichuan University, Chengdu, Sichuan 610041, People’s Republic of China.; 5 Zhong Hui Wen, MD, Department of Gastroenterology, West China Hospital, Sichuan University, Chengdu, Sichuan 610041, People’s Republic of China.

**Keywords:** Abdominal pain, Hereditary angioedema, C1-INH

## Abstract

Hereditary angioedema is a rare autosomal dominant inherited disease which is characterized by an episodic, self-limiting increase in vascular permeability. Symptoms commonly involve in nonpitting, nonpruritic skin swellings. We present a case of hereditary angioedema. The patinets complained of a recurrent abdominal pain without accompanying skin swelling whose diagnosis was delayed nearly 20 years and accepted an unnecessary surgery. According to the decreased serum C1-inhibitor and C4 concentration, the patient was finally diagnosed with hereditary angioedema type I. After treatment with danazole, the patient reported a significant decrease in the frequency of attacks and the severity of pain. HAE is a rare cause of abdominal pain, however it needs to be taken as one of the differential diagnosis of various acute abdomens in order to avoid unnecessary surgeries.

## INTRODUCTION

Hereditary angioedema (HAE) is a rare autosomal dominant inherited disease that disturbs approximately one in every 10000 to 50000 individuals worldwide.^1^ HAE is characterized by episodic, self-limiting edema of the subcutaneous and submucosal tissues. Skin is usually involved, though not common, respiratory or gastrointestinal tract suffering was also reported.^2^ When localized in the gastrointestinal tract, it can cause severe abdominal pain, mimicking an acute surgical abdomen and could lead to unnecessary surgeries.

## CASE REPORT

A 50-year-old woman presented with long episodes of abdominal pain with no apparent inducement over the past 20 years. At the third time of the acute abdominal attack, the patient underwent an appendisectomy, but similar symptoms still troubled her contineously. Interestingly, she had past history of occasional nonpitting, nonpruritic swellings in her bodies, but the hydroderma was not synchronized with the abdominal pain and seldom occurred in recent years. Additional history indicated an allergy to penicillin and seafood. Her father and one of her two sisters had similar complaints with skin edema but both without the abdominal pain, while other family members never suffered these pains.

**Fig.1:**
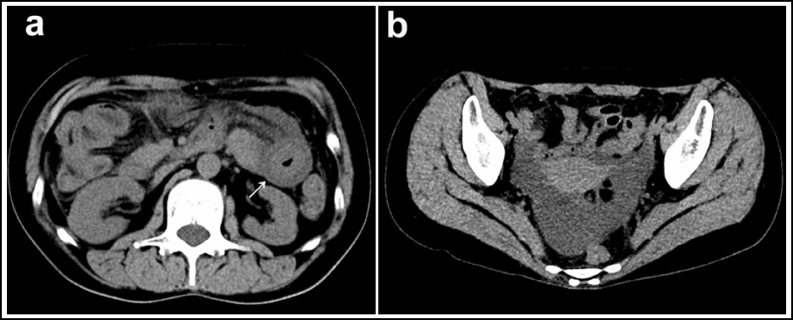
CT images of the patient. Computed tomography scan of the abdomen showing circumferential edema of the proximal jejunal bowel (arrow-head) and significant thicken of the mesenterium (a), together with a moderate amount of ascites (b).

A physical examination, blood chemistry analysis didn’t reveal anything abnormal. Immunologic tests never retrieved pathologic finding except C3 and C4. Computed tomography (CT) scan of the abdomen showed circumferential edema of the proximal jejunal bowel with thickened mesentery. A moderate amount of ascites was also detected (Fig.1). Several erythemas were seen in views on gastroscopy, colonoscopy and capsule endoscopy tests.

During the past 8 years, she had been classified as having appendicitis, pancreatitis, ischemic enteropathy or irritable bowel syndrome, and treated with H1-receptor antagonists, glucocorticoids, cholinoceptor blocking drugs, etc. Though symptoms could relieve after above treatments for two or three days, seizure frequency and severity seemed to keep worsening from yearly to trimestrally and even to monthly in recent two years.

In an effort to investigate the cause of the curious abdominal pain and ascites, we carefully reviewed the history and related literatures. Then, the positive family history of hydroderma and the unexplained low level of C4 attracted our attention. Thus, HAE was taken into consideration. The level of serum C1-inhibitor (C1-INH) concentration was evaluated in Laboratory of Peking Union Medical College Hospital. As shown in Table-I, the serum concentration of C1-INH demonstrated a significant reduction.

According to the decreased serum C1-INH and C4 concentration, the patient was finally diagnosed with HAE type I. After she accepted 600mg/d danazole treatment, our patient reported a significant decrease in the frequency of attacks and the severity of pain. After two month of treatment, the doses were reduced by half, and the symptoms kept relieving during the following six months. The patient seldom developed critical side effect, except for weight gain over the two-month treatment process.

## DISCUSSION

Patient who presents with gastrointestinal symptoms of HAE without skin edema is rare and can be hard to identify during a host of acute abdomens as in this case. Nearly one-quarter of patients with HAE presenting to emergency departments meet lab diagnosis or misdiagnosis^3^ that obviously increases risk of death or disability. Thus, in patients with unexplained acute attacks of abdominal pain, though not common, HAE should be taken into consideration.

**Table-I:** Results of patient’s C3, C4 and C1-INH

	***Normal***	***Patient***
C1-INH (g/L)	0.05	0.21-0.3
C3 (g/L)	0.6910	0.785-1.52
C4 (g/L)	0.0329	0.145-0.36

HAE is clinically manifested by recurrent episodes of localized, nonpitting, nonpruritic, subcutaneous or submucosal edema lasting for 2-5 days. The possible mechanism of these clinical manifestations is that C1-INH deficiency or dysfunction which is due to mutations in C1-INH gene, leads to uncontrolled activation and enhanced auto-activation of the complement system, resulting in elevated bradykinin levels. These high levels of bradykinin increase vascular permeability, cause vasodilatation and smooth muscle contraction, which can lead to edema and pain.^4^^,^^5^

According to the levels of C4 and C1-INH, HAE can be classified into two different types traditionally. HAE type I, accounting for 85% of HAE patients, is characterized by decreased serum level of C1-INH and C4. In contrast, HAE type II patients (15%) show normal or increased but dysfunctional C1-INH with reduced C4 level.^6^ Notably, HAE type III was recently described with normal C1-INH and C4 concentrations in women.^7^ In our case, the low level of C1-INH and C4 ruled out HAE type I.

Treatment of HAE involves acute attacks release and long-term prophylaxis. An emergency treatment with the plasma-derived or recombinant C1-INH administered by intravenous infusion at a dose of 20 U/kg can induce an unusually dramatic regression of all symptoms in 30 to 60 minutes.^8^ Kallikrein inhibitor ecallantide (Kalbitor), and a bradykinin B2 receptor antagonist icatibant (Firazyr) are also approved for the treatment of acute episodes in recent years. For long-term prophylaxis, the attenuated androgens (danazol and stanozolol) are recommended since they can improve the level of serum C4 concentration and the synthesis of C1-INH. Both clinical efficacy and side effect of long-term prophylaxis with androgen derivatives in HAE are dose dependent, recommended doses with acceptable adverse effects are danazol≤200mg/d and stanozolol≤2mg/d.^8^ Conventional drugs such as antihistamines, glucocorticoids and epinephrine are not effective in most reports,^9^ but convincing clinical trials are required to confirm the efficacy of these drugs in HAE. 

## Authors contribution:

**Xi Chen** participated in collecting the data and writing the manuscript.

**Ying Xue Yang and Yu Lan Liu **were involved in follow-up.

**Zhong Hui Wen** **and Hua Tian Gan** participated in revising the manuscript.

All authors have read and approved the final manuscript.

## Notes

***Source of funding: ***None.
